# The Law as It Relates to the Physicians and the Buffalo Health Department

**Published:** 1912-05

**Authors:** Francis E. Fronczak

**Affiliations:** Health Commissioner; Buffalo, Addendum


### The Law as it Relates to the Physicians and the Buffalo Health Department.

Dr. FRANCIS E. FRONCZAK, Health Commissioner, Buffalo, Addendum.

The following paragraph is to be inserted above the next to the last paragraph on Page 479, April issue, and to follow the sentence which ends as follows:

"That would preclude such embalming from a medico-legal standpoint."

If dead bodies are to be cremated instead of buried, an affidavit from the attending physician or Medical Examiner must accompany the death certificate. This affidavit must state that the physician or examiner is acquainted with all the facts and circumstances connected with the death of the deceased, and that, to the best of his knowledge, there exists no reason why the body should not be cremated.

Note: We expect soon to publish a second communication from Dr. Fronczak on the Law Regarding Insanity.

